# Comprehensive Assessment of Cyclic Fatigue Strength in Five Multiple-File Nickel–Titanium Endodontic Systems

**DOI:** 10.3390/ma17102345

**Published:** 2024-05-15

**Authors:** Jorge N. R. Martins, Emmanuel J. N. L. Silva, Duarte Marques, Francisco M. Braz Fernandes, Marco A. Versiani

**Affiliations:** 1 Faculdade de Medicina Dentária, Universidade de Lisboa, 1600-277 Lisboa, Portugal; 2 LIBPhys-FCT UID/FIS/04559/2013, 1600-277 Lisboa, Portugal; 3 Grupo de Investigação em Bioquimica e Biologia Oral (GIBBO), Unidade de Investigação em Ciências Orais e Biomédicas (UICOB), 1600-277 Lisboa, Portugal; 4 Centro de Estudos de Medicina Dentária Baseada na Evidência (CEMDBE), 1600-277 Lisboa, Portugal; 5 Department of Endodontics, School of Dentistry, Grande Rio University (UNIGRANRIO), Rio de Janeiro 21210-623, Brazil; 6 Department of Endodontics, Fluminense Federal University, Rio de Janeiro 24220-900, Brazil; 7 Department of Endodontics, Rio de Janeiro University (UERJ), Rio de Janeiro 20550-013, Brazil; 8 CENIMAT/I3N, Department of Materials Science, NOVA School of Science and Technology, Universidade NOVA de Lisboa, 2829-516 Caparica, Portugal; 9 Dental Specialty Center, Brazilian Military Police, Belo Horizonte 30350-190, Brazil

**Keywords:** cyclic fatigue, differential scanning calorimetry, endodontics, energy-dispersive X-ray spectroscopy, root canal treatment

## Abstract

The resistance of nickel–titanium endodontic instruments against cyclic fatigue failure remains a significant concern in clinical settings. This study aimed to assess the cyclic fatigue strength of five nickel–titanium rotary systems, while correlating the results with the instruments’ geometric and metallurgical characteristics. A total of 250 new instruments (sizes S1/A1, S2/A2, F1/B1, F2/B2, F3/B3) from ProTaper Gold, ProTaper Universal, Premium Taper Gold, Go-Taper Flex, and U-Files systems underwent mechanical testing. Prior to experimental procedures, all instruments were meticulously inspected to identify irregularities that could affect the investigation. Using a stereomicroscope, design characteristics such as the number of spirals, length, spirals per millimeter, and average helical angle of the active blade were determined. The surface finishing characteristics of the instruments were examined using a scanning electron microscope. Differential scanning calorimetry was employed to establish the instruments’ phase transformation temperatures, while energy-dispersive X-ray spectroscopy was utilized to analyze the elemental composition of the alloy. The instruments were subjected to cyclic fatigue testing within a stainless steel non-tapered artificial canal featuring a 6 mm radius and 86 degrees of curvature. Appropriate statistical tests were applied to compare groups, considering a significance level of 0.05. The assessed design characteristics varied depending on the instrument type. The least irregular surface finishing was observed in U-Files and Premium Taper Gold files, while the most irregular surface was noted in Go-Taper Flex. All instruments exhibited near-equiatomic proportions of nickel and titanium elements, whereas ProTaper Universal and U-Files instruments demonstrated lower phase transformation temperatures compared to their counterparts. Larger-sized instruments, as well as ProTaper Universal and U-Files, tended to display lower cyclic fatigue strength results. Overall, the design, metallurgical, and cyclic fatigue outcomes varied among instruments and systems. Understanding these outcomes may assist clinicians in making more informed decisions regarding instrument selection.

## 1. Introduction

Root canal therapy is a fundamental aspect of modern dentistry, aiming to relieve pain and preserve natural teeth [1]. Critical for the success of these procedures is the use of endodontic files, which play a vital role in thoroughly shaping and cleaning the root canal space [1]. Over time, there has been a significant transformation in the approach to root canal preparation [1]. Initially reliant on stiff stainless steel hand files, modern techniques have embraced the use of nickel–titanium (NiTi) instruments [1]. These instruments offer remarkable advancements, including superior flexibility, heightened resistance to torsional stress, and an overall enhancement in performance [1]. However, the potential susceptibility of NiTi instruments to cyclic fatigue failure still remains a significant concern in clinical practice [1]. Cyclic fatigue failure occurs when microcracks form and accumulate due to repeated stress cycles [2]. This phenomenon primarily occurs at points of maximum flexure within a specific curved root canal, potentially resulting in instrument separation or other procedural errors that may compromise treatment outcomes [1,3]. Therefore, the evaluation of cyclic fatigue strength of NiTi systems becomes critically important for assessing instrument performance.

To understand the cyclic fatigue performance of endodontic instruments, a consistent and comprehensive testing methodology is required [4]. Various factors, such as radius and degree of curvature, temperature, rotational speed, and kinematic or axial motion, can influence the fatigue strength of NiTi instruments [5,6,7]. Therefore, standardized testing protocols have been devised to quantify cyclic fatigue strength, enabling comparative analysis among different systems [6]. The significance of testing cyclic fatigue strength lies in its direct correlation with clinical efficacy and patient safety [6]. Endodontic instruments subjected to cyclic fatigue are susceptible to structural deformation and microfractures, compromising their cutting efficiency and increasing the risk of fracture within the root canal [1,2,3]. Consequently, the likelihood of procedural errors, canal transportation, and instrument separation rises, potentially leading to treatment failure and potentially requiring a retreatment approach [1,3]. Moreover, separated fragments of endodontic instruments represent a significant challenge in root canal retreatment, often requiring complex retrieval techniques and extending treatment time and cost [8].

The variability in cyclic fatigue strength outcomes across different NiTi systems highlights the importance of evidence-based selection to optimize clinical outcomes [9]. Although manufacturers strive to enhance the fatigue strength of NiTi instruments through advancements in alloy characteristics and manufacturing processes, clinicians must carefully assess the mechanical performance of the numerous systems available on the market [9]. By systematically evaluating cyclic fatigue strength, clinicians can make more informed decisions regarding instrument selection for specific clinical cases, ultimately enhancing the predictability and success of root canal treatment.

The present study aims to perform a comprehensive assessment of the cyclic fatigue strength of five multiple-file NiTi systems, encompassing a total of 25 different endodontic instruments, and to correlate these results with their geometric and metallurgical characteristics. The null hypothesis under examination was that there would be no differences in cyclic fatigue strength among similar instruments from different brands.

## 2. Materials and Methods

The study evaluated the geometric design, metallurgical properties, and cyclic fatigue performance of 25 mm NiTi instruments from five different rotary system brands (Table 1): ProTaper Gold (PTG; Dentsply, Ballaigues, Switzerland); ProTaper Universal (PTU; Dentsply, Ballaigues, Switzerland); Premium Taper Gold (Waldent, Shenzhen, China); Go-Taper Flex (Access, Shenzhen, China); and U-File (Dentmark, Ludhiana, India). A total of 250 instruments underwent mechanical testing.

### 2.1. Geometric Design Analysis

Using a stereomicroscope (Opmi Pico, Carl Zeiss Surgical, Germany) with magnifications of ×3.4 and ×13.6, six randomly selected instruments of each type underwent examination. The parameters evaluated included the following: count of spirals on the active blade, length of the active blade in millimeters, spirals per millimeter on the active blade, average helical angle of six coronal angles of the active blade, and identification of major defects or deformations such as missed, twisted, or distorted blades. Subsequently, conventional scanning electron microscopy (SEM) (S-2400, Hitachi, Tokyo, Japan) at ×500 magnification was used to examine the same instruments for surface marks resulting from manufacturing processes and minor manufacturer deformations or defects.

### 2.2. Metallurgical Features Analysis

Semi-quantitative elemental analysis was conducted using energy-dispersive X-ray spectroscopy and scanning electron microscopy (EDS/SEM), while differential scanning calorimetry (DSC) was employed to examine the phase transformation temperatures of the instruments. EDS/SEM analysis was carried out on the surface (400 µm^2^) of three F1(B1) instruments of each file system, with an SEM device (S-2400, Hitachi) set at 20 kV and 3.1 amperes connected to an EDS detector (Bruker Quantax, Bruker Corporation, Billerica, MA, USA), employing dedicated software with ZAF correction (Systat Software Inc., San Jose, CA, USA) for analysis. DSC analysis (DSC 204 F1 Phoenix; Netzsch-Gerätebau GmbH, Selb, Germany) was performed on two different F1(B1) instruments of each system in accordance with the American Society for Testing and Materials (ASTM) F2004-17 guidelines [10]. A 3 to 5 mm fragment (7 to 10 mg) obtained from the coronal active portion of each instrument underwent chemical etching in a mixture of 45% nitric acid, 30% distilled water, and 25% hydrofluoric acid for 2 min, followed by mounting in an aluminum pan. Thermal cycling was conducted with temperatures ranging from 150 °C to −150 °C (cooling/heating rate: 10 K/min) under a nitrogen (N_2_) atmosphere. DSC charts for visual analysis of transformation temperatures were generated using Netzsch Proteus thermal analysis software (Netzsch-Gerätebau GmbH V7.1). Each group underwent the DSC test twice as a validation measure to ensure the accuracy and reliability of the initial findings.

### 2.3. Cyclic Fatigue Testing

Before starting the cyclic fatigue experimental procedure, all instruments underwent a visual inspection using stereomicroscopy (×13.6 magnification) to identify any deformities or defects that might render them ineligible for the study. No deformities were observed during this examination. The F1/B1 instrument was selected as the reference for determining the sample size, taking into account a power of 80% and an alpha-type error of 0.05. This calculation was derived from the largest observed difference among different systems observed during six initial tests, which, in this case, was the comparison between PTU and Premium Taper Gold systems. For the time to fracture analysis, an effect size of 161.5 ± 100.5 (PTU vs. Premium Taper Gold) determined a requirement of 8 instruments per group. To compensate for the absence of sample size calculation for the S1/A1, S2/A2, F2/B2, and F3/B3 instruments, the number of instruments for each test was increased to 10 to maintain balance.

Ten instruments of each type (S1/A1, S2/A2, F1/B1, F2/B2, and F3/B3) were mounted onto a 6:1 reduction handpiece (VDW/Sirona Dental Systems, Bensheim, Germany), which was operated by a torque-controlled motor (VDW Silver; VDW GmbH, Munich, Germany). This handpiece was connected to a custom-made tube model device (Odeme Dental Research, Luzerna, Santa Catarina, Brazil), ensuring uniform testing conditions for all instruments. The instruments were subjected to continuous clockwise rotation within a stainless steel non-tapered artificial canal with a 6 mm radius and 86 degrees of curvature, measuring 9 mm in length. The point of maximum stress was located at the midpoint of the curved section (around 7 mm from the tube terminal point). The cyclic fatigue test was conducted in a static model, with glycerin lubrication at room temperature (20 °C), adhering to the rotational motion specified by the manufacturer [11]. The instruments rotated freely within the artificial canal until a fracture occurred, which was detected visually and audibly. The time elapsed until fracture was recorded using a digital chronometer, and the size of the fractured fragment was measured with a digital caliper (Mitutoyo, Aurora, IL, USA).

### 2.4. Statistical Analysis

The Shapiro–Wilk test was used to evaluate the assumption of normality in the variance of the time to fracture results. For data with a normal distribution, comparisons were conducted using one-way ANOVA followed by post hoc Tukey tests. In instances where the data did not conform to a Gaussian distribution, the non-parametric Mood’s median test was employed. The data outcomes were summarized using median and interquartile range. A significance level of 5% was set (SPSS v22.0 for Windows; SPSS Inc., Chicago, IL, USA).

## 3. Results

### 3.1. Geometric Design Analysis

**Figure 1:**
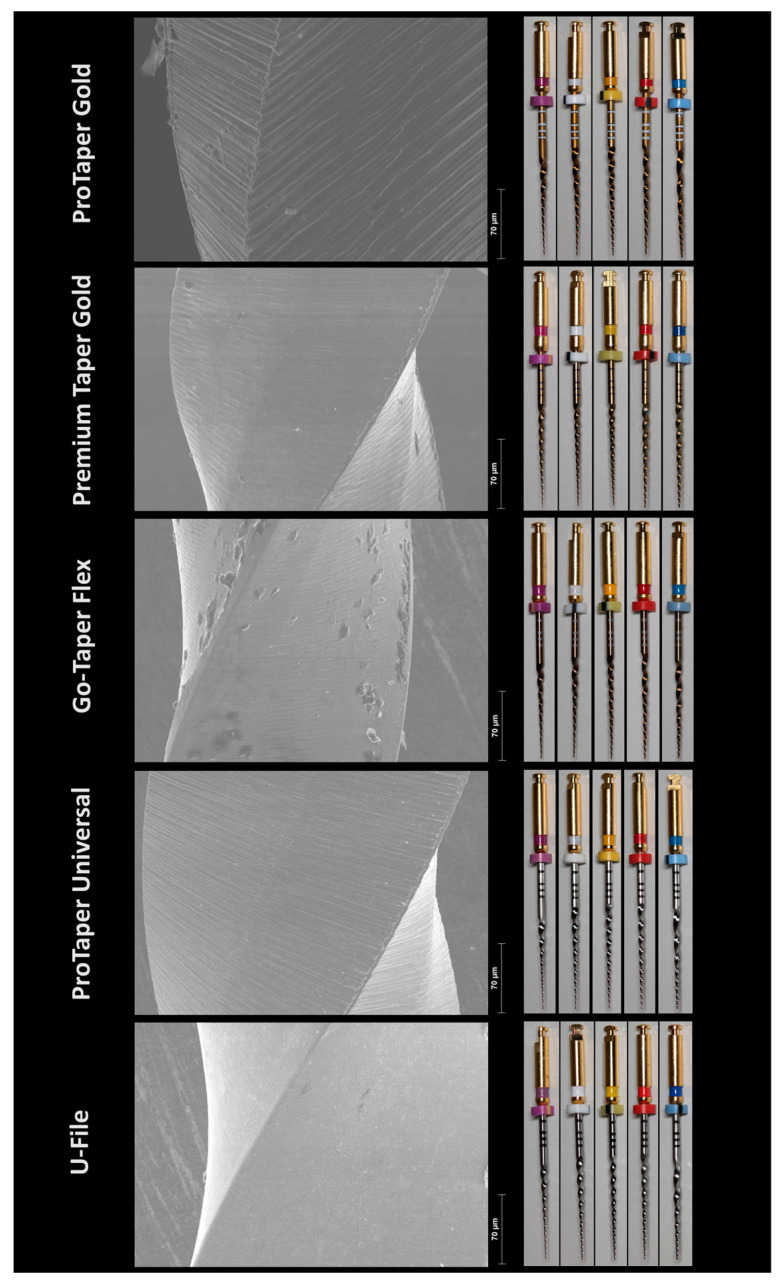
Geometric characteristics of the assessed instruments. On the left side, the SEM analysis (magnification: 500×; high voltage: 20.0 kV) illustrates the surface finishing of the instruments, with the Go-Taper Flex group displaying a more irregular surface. On the right side, the tested instruments from multiple systems are shown, with colors indicating different types (S1/A1 in purple, S2/A2 in white, F1/B1 in yellow, F2/B2 in red, and F3/B3 in blue).

The geometric design characteristics are illustrated in Figure 1 and summarized in Table 1. The active blade length remained consistent at 17 mm for instruments sizes S2/A2, F1/B1, and F2/B2, whereas it ranged from 15 mm (for ProTaper Gold, ProTaper Universal, and U-File) to 17 mm (for Premium Taper Gold) in S1/A1 instruments. The number of spirals varied across instrument sizes, with F1/B1 exhibiting the highest number and F3/B3 the lowest.

Premium Taper Gold instruments consistently displayed a higher number of spirals across all instrument sizes, with an additional tendency to exhibit a superior number of spirals per millimeter and helical angles. The SEM inspection revealed a smoother surface finish in both U-File and Premium Taper Gold instruments, whereas both ProTaper Gold and Universal exhibited the most visible parallel surface marks, consistent with the machining process (Figure 1). The Go-Taper Flex instruments displayed a most irregular surface pattern (Figure 1).

### 3.2. Metallurgical Features Analysis

**Figure 2:**
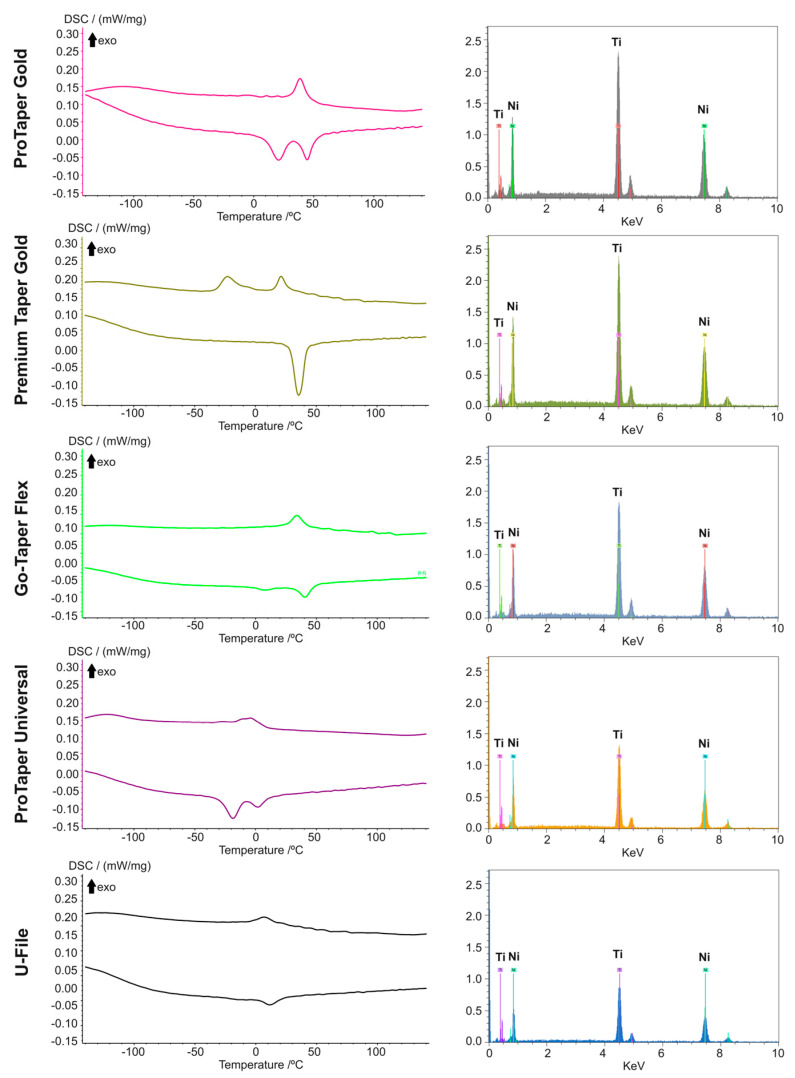
Metallurgical characteristics of the tested groups. On the left side, the phase transformation temperatures indicate higher values for ProTaper Gold, Premium Taper Gold, and Go-Taper Flex, and lower values for the ProTaper Universal and U-File systems. On the right side, the elemental composition analysis reveals comparable features across all groups.

Figure 2 and Table 2 provide a summary of the metallurgical characteristics of the evaluated systems. The elemental composition analysis revealed that all systems were made of NiTi alloy with nearly equiatomic proportions of nickel and titanium, without any traces of other metallic elements. The phase transformation temperature analysis indicated higher R-phase start (Rs) (49.3 °C and 43 °C) and R-phase finishing (Rf) (30.7 °C and 23.8 °C) temperatures in ProTaper Gold and Go-Taper Flex files, respectively. Conversely, ProTaper Universal files exhibited the lowest Rs (10.1 °C), Rf (−16.9 °C), austenitic start (As) (−30.2 °C), and austenitic finish (Af) (12.1 °C) temperatures.

### 3.3. Cyclic Fatigue Testing

**Figure 3:**
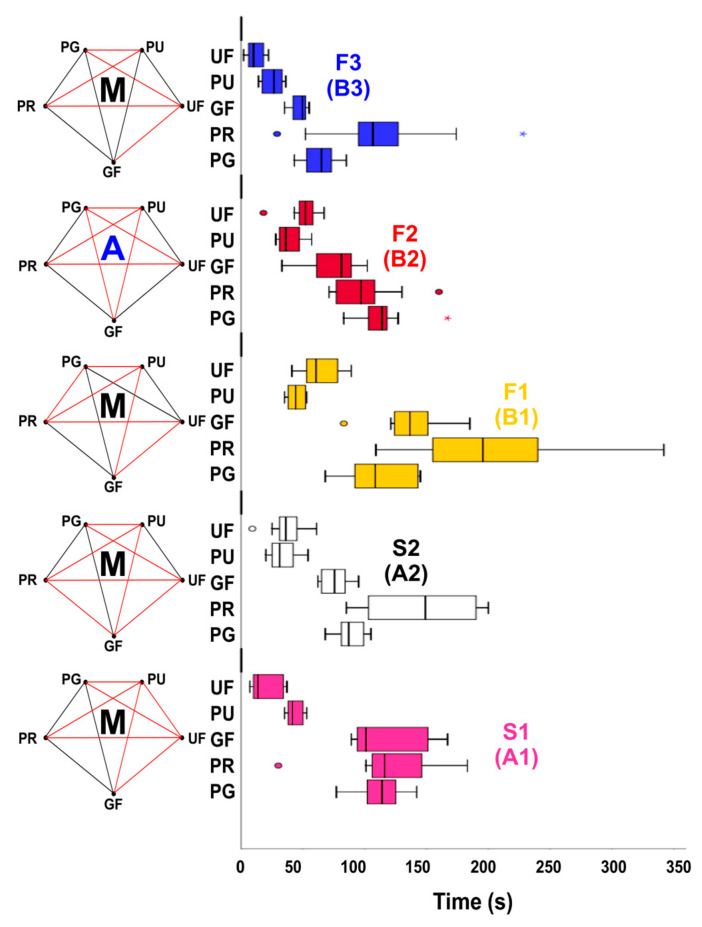
The results of the cyclic fatigue testing indicate that larger-sized instruments (F2/B2 and F3/B3), as well as ProTaper Universal (PTU) and U-Files (UF), tended to show lower outcomes (PG: ProTaper Gold; PR: Premium Taper Gold; GF: Go-Taper Flex; M: Mood’s median test; A: ANOVA test).

The results of the cyclic fatigue test are presented in Figure 3 and Table 1. The findings indicated that larger instruments (sizes F2/B2 and F3/B3) exhibited shorter time to fracture compared to instruments with smaller dimensions. Instruments from the ProTaper Universal and U-Files systems consistently yielded lower results for all instruments (*p* < 0.05), while the Premium Taper Gold system consistently displayed the highest time to fracture (*p* < 0.05). The size of the fractured fragments remained consistent across all instruments, ranging from 7.1 mm to 8.4 mm.

## 4. Discussion

Endodontic instruments are indispensable tools in root canal treatment, but their susceptibility to cyclic fatigue failure remains a challenge. Therefore, it is essential to comprehend the outcomes of fatigue tests from both geometric and metallurgical viewpoints in order to enhance instrument durability and clinical efficacy, while also helping to uncover potential causes of premature failure [12,13]. The design of the instruments influences stress distribution and cyclic fatigue strength. Factors such as size and taper, cross-section geometry, number of cutting spirals, and debris removal efficiency can significantly impact outcomes [14,15,16,17]. Optimizing these parameters may enhance fatigue strength and prolong the clinical longevity of instruments. The composition of instrument elements and processing methods can also affect cyclic fatigue strength. Metal alloy composition, grain structure, and heat treatment techniques are important in determining material strength under cyclic loading [18,19,20,21]. Studying microstructural changes and deformation mechanisms under cyclic stress aids in optimizing the manufacturing process and selecting instruments for clinical use. In this way, the interaction between the geometric design and metallurgical properties of endodontic instruments is crucial. Optimizing both geometric characteristics and material properties synergistically enhances instrument performance. Understanding this interplay can lead to the development of more robust instruments capable of withstanding cyclic loading with reduced risk of failure.

Although the stress applied to an instrument during clinical use is not solely due to cyclic fatigue loading, it is expected that enhancing cyclic fatigue strength will translate into a longer instrument lifespan, decreased risk of file separation, and improved treatment efficiency. Insights gained from research can aid clinicians in making better-informed decisions regarding instrument selection, usage, and maintenance, ultimately leading to superior clinical outcomes for patients. The present research observed significant differences among instruments of similar sizes from different brands, thus rejecting the null hypothesis.

The DSC results confirm the manufacturer’s claim that ProTaper Gold, Premium Taper Gold, and Go-Taper Flex files were produced using heat treatment procedures. This helps explain their superior performance compared to equivalent instruments from ProTaper Universal and U-File systems, which appear to be manufactured with conventional NiTi alloys [22,23]. Overall, Premium Taper Gold instruments exhibited significantly higher cyclic fatigue strength, a finding that may be partly attributed to their heat treatment, smoother surface finishing [14], and greater number of spirals and spirals per millimeter in most of their instruments, which are features that tend to increase the cyclic fatigue strength [17]. However, it is noteworthy that while a longer clinical lifespan is expected, the most optimal clinical performance depends on achieving a balance between flexibility and torsional strength. A previous study [24] addressing this NiTi system also reported instances of severe plastic deformation and fractures during mechanical preparation of canals in extracted teeth, highlighting potential clinical drawbacks associated with excessively flexible instruments. The lower cyclic fatigue results among the heat-treated systems were observed with Go-Taper Flex files, which may be partly explained by their increased surface irregularities favoring the initiation of cracks [2,14]. Another trend noted in this research is that larger-sized instruments, such as F2/B2 and F3/B3 (their larger sizes are well exposed in the apical sizes documented in Table 1 and the instruments profile, which can be observed in Figure 1), exhibited reduced fatigue strength, consistent with findings from previous studies [16,25,26].

In this study, the cyclic fatigue test was conducted at room temperature (20 °C). Currently, there is no consensus on the optimal testing temperature for evaluating endodontic NiTi instruments as they typically operate within a service temperature range of 20 °C to 36 °C rather than at a specific, constant temperature [6]. The factors contributing to temperature changes in instruments during root canal treatment are multifaceted, making it impractical to account for all of them in a single testing setup. Therefore, room temperature (20 °C) was chosen as the testing temperature, aligning with ASTM F2516-07 guidelines [27] and a proposed update [28] to the ISO 3630-1 standard [28,29]. Additionally, a phase transformation temperature assessment was conducted to comprehensively understand potential changes in instrument performance at different temperatures, using the baseline results collected at room temperature as a reference. The DSC heating charts indicated that ProTaper Universal and U-File exhibit an austenitic crystallographic arrangement within the instruments’ service temperature range. Both ProTaper Gold and Go-Taper Flex showed a mixture of R-Phase and austenitic within the same range. Conversely, the assessment of the Premium Taper Gold system revealed a transition in the crystallographic arrangement from R-Phase to a mixture of R-Phase and austenitic. This suggests that while all systems may experience minor performance changes due to temperature increases, the Premium Taper Gold system is potentially the most affected due to the presence of a phase transformation within the service temperature range. In this case, such a transformation may lead to a decrease in cyclic fatigue strength attributed to the addition of the austenitic phase to its crystallographic arrangement.

One limitation of this study is the lack of other mechanical tests to corroborate the current findings, such as torsional and bending testing. However, performance data of these instruments has been previously reported (24), aligning with the flexibility findings of the present study through alternative tests. Moreover, it is important to recognize that cyclic fatigue alone represents the strength of the metal alloy under a very specific type of stress, which may not fully capture the complexity of clinical practice. Consequently, the conclusions drawn from this investigation should be restricted to the results of this particular test. A strength of the present study is related to the previous absence of cyclic fatigue data regarding the Premium Taper Gold, Go-Taper Flex, and U-File systems. While the study demonstrates high internal validity due to its meticulous and reproducible methodology, caution should be exercised when interpreting the external validity and generalizing the results, necessitating additional data from other research perspectives to develop a more comprehensive understanding. Microstructural analysis of the nickel–titanium files before and after the cyclic fatigue tests would also provide revealing insights into the failure mechanisms.

## 5. Conclusions

The findings of the present study highlight the variability in geometric design, metallurgical features, and cyclic fatigue strength among different endodontic instruments and systems. Small-sized instruments and those made of heat-treated NiTi alloys demonstrated superior cyclic fatigue strength. Within the heat-treated category, Premium Taper Gold instruments tended to display the highest time to fracture, while Go-Taper Flex instruments exhibited the lowest. These results underscore the importance of considering both geometric and metallurgical factors when assessing the performance of endodontic instruments as they can significantly influence their durability and effectiveness in clinical practice.

## Figures and Tables

**Table 1:** Assessed instruments’ geometric design characteristics and results of time to fracture and separated fragment length presented as the median [interquartile range].

Instruments	Geometric Design					Cyclic Fatigue Test ^1^	
	Apical Size/Taper ^2^	Active Blade Length (mm)	Number of Spirals	Spirals per Millimetre	Helical Angle (°)	Time to Fracture (s)	Fragment Length (mm)
ProTaper Gold S1	18/0.02 v	15	11	0.73	21.7°	114 [99–128]	8.0 [7.6–8.1]
Premium Taper Gold S1	18/0.02 v	17	12	0.71	24.3°	116 [104–148]	7.4 [7.4–7.5]
Go-Taper Flex A1	18/0.02 v	16	11	0.69	19.6°	101 [93–154]	7.7 [7.4–7.9]
ProTaper Universal S1	17/0.02 v	15	11	0.73	21.6°	41 [37–50]	7.5 [6.9–7.7]
U-File S1	17/0.02 v	15	11	0.73	20.3°	13 [10–34]	7.5 [7.2–7.9]
ProTaper Gold S2	20/0.04 v	17	11	0.65	22.4°	87 [80–100]	7.7 [7.4–8.2]
Premium Taper Gold S2	20/0.04 v	17	14	0.82	29.6°	149 [102–192]	7.7 [7.4–7.8]
Go-Taper Flex A2	20/0.04 v	17	11	0.65	23.6°	75 [65–86]	7.8 [7.4–7.9]
ProTaper Universal S2	20/0.04 v	17	11	0.65	22.3°	31 [24–42]	7.2 [6.9–7.8]
U-File S2	20/0.04 v	17	11	0.65	21.4°	36 [29–47]	7.3 [7.0–7.8]
ProTaper Gold F1	20/0.07 v	17	12	0.71	24.7°	108 [89–143]	7.6 [7.0–7.9]
Premium Taper Gold F1	20/0.07 v	17	15	0.88	29.7°	186 [140–236]	7.9 [7.7–8.0]
Go-Taper Flex B1	20/0.07 v	17	12	0.71	25.8°	136 [123–151]	8.0 [7.4–8.2]
ProTaper Universal F1	20/0.07 v	17	12	0.71	25.4°	44 [38–52]	7.8 [7.6–8.0]
U-File F1	20/0.07 v	17	12	0.71	24.4°	60 [52–79]	7.3 [7.1–7.6]
ProTaper Gold F2	25/0.08 v	17	10	0.59	22.0°	114 [100–120]	8.3 [7.6–8.9]
Premium Taper Gold F2	25/0.08 v	17	11	0.65	25.1°	97 [76–113]	7.9 [7.7–8.1]
Go-Taper Flex B2	25/0.08 v	17	10	0.59	23.3°	81 [59–90]	7.7 [7.5–8.4]
ProTaper Universal F2	25/0.08 v	17	10	0.59	22.3°	36 [30–47]	7.1 [6.9–7.5]
U-File F2	25/0.08 v	17	11	0.65	25.6°	52 [46–58]	7.3 [6.3–8.2]
ProTaper Gold F3	30/0.09 v	17	9	0.53	21.5°	65 [52–74]	8.4 [7.8–8.6]
Premium Taper Gold F3	30/0.09 v	17	11	0.65	27.4°	106 [84–138]	8.1 [6.5–10.4]
Go-Taper Flex B3	30/0.09 v	16	9	0.56	22.1°	49 [40–52]	8.4 [7.9–8.9]
ProTaper Universal F3	30/0.09 v	17	9	0.53	21.6°	26 [16–33]	8.1 [7.5–9.8]
U-File F3	30/0.09 v	16	9	0.56	23.1°	10 [5–18]	8.2 [7.2–9.4]

^1^ Figure 3 summarizes the statistical differences between instruments. ^2^ Information according to the manufacturer.

**Table 2:** Phase transformation temperatures and elements composition of the reference instruments.

Instruments *	Phase Transformation Temperatures				Elements Composition		
	Rs °C Cooling	Rf °C Cooling	As °C Heating	Af °C Heating	Nickel (Atomic %)	Titanium (Atomic %)	Nickel/Titanium Ratio
ProTaper Gold F1	49.3 °C	30.7 °C	8.9 °C	51.3 °C	51.58	48.42	1.065
Premium Taper Gold F1	26.6 °C	16.5 °C	27.5 °C	42.8 °C	51.20	48.80	1.049
Go-Taper Flex B1	43.0 °C	23.8 °C	−0.8 °C	49.1 °C	50.72	49.28	1.029
ProTaper Universal F1	10.1 °C	−16.9 °C	−30.2 °C	12.1 °C	50.47	49.53	1.019
U-File F1	18.7 °C	−4.4 °C	3.6 °C	24.8 °C	50.23	49.77	1.009

* Only the F1(B1) instruments were submitted for DSC and EDS testing and considered as references for each one of the assessed file systems. Rs: R-phase start; Rf: R-phase finish; As: Austenitic start; Af: Austenitic finish.

## Data Availability

Data are contained within the article.
